# Pools versus Queues: The Variable Dynamics of Stochastic “Steady States”

**DOI:** 10.1371/journal.pone.0130574

**Published:** 2015-06-19

**Authors:** Eric T. Lofgren

**Affiliations:** Network Dynamics and Simulation Science Laboratory, Virginia Bioinformatics Institute Virginia Tech, Blacksburg, Virginia, United States of America; Arizona State University, UNITED STATES

## Abstract

Mathematical models in ecology and epidemiology often consider populations “at equilibrium”, where in-flows, such as births, equal out-flows, such as death. For stochastic models, what is meant by equilibrium is less clear – should the population size be fixed or growing and shrinking with equal probability? Two different mechanisms to implement a stochastic steady state are considered. Under these mechanisms, both a predator-prey model and an epidemic model have vastly different outcomes, including the median population values for both predators and prey and the median levels of infection within a hospital (*P* < 0.001 for all comparisons). These results suggest that the question of how a stochastic steady state is modeled, and what it implies for the dynamics of the system, should be carefully considered.

## Introduction

Dynamic models have a long and well-established history of providing insight into biological systems by allowing the synthesis of disparate empirical data into cohesive theory[1,2]. One of the most common ways of formulating these dynamic models is with the use of differential equations. Such models enjoy the potential for an analytic solution and the widespread availability of numerical toolkits for solving even extremely complex systems of equations computationally.

These models are not without their drawbacks. They model populations continuously, rather than discretely, which often results in populations within the model being reported in small fractional numbers at the system asymptotically approaches zero. For large populations, this tendency is of no concern, as 0.01 percent of the U.S. population, for example, is still a meaningful number. For small populations however, this tendency becomes extremely problematic, as the model begins to report the existence of fractional individuals, a biologically meaningless concept. This phenomenon, known as the “atto-fox problem” [3], makes these purely deterministic, continuous scale models inappropriate for use in small populations[4]. Biologically, there can be one or more foxes or no foxes, but the population cannot be 10^−18^ foxes.

One solution to this problem has been to use a stochastic simulation approach with discrete values for the population[5]. This approach allows for the direct adaptation of the differential equation based models into a stochastic framework. Not only does this approach more realistically model integer-valued individuals, but also it allows for stochastic extinction–the complete loss of one population from the model due to random demographic noise. In contrast, a differential equation based model will always maintain some vanishingly small residual population that may some day rebound.

Stochastic extinction can even arise in populations that are being modeled as being in a steady state, where in-flows (i.e. births and immigration) are equal to out-flows (i.e. deaths and emigration), representing a population at equilibrium save for the dynamic process the researcher is exploring. How one approaches formulating the stochastic simulation has a profound impact on the dynamics of the system as a whole. Here, I present two different formulations of steady state for a model that have identical outcomes when modeled using differential equations, but have vastly different outcomes when simulated stochastically. I have termed these “Pool” and “Queue” stability. I also suggest there are not one but two types of stochastic extinction present in most stochastic dynamic models: *C-extinction* (the extinction of a given group within the model) and *N-extinction* (the extinction of the entire model population), either one of which may be potentially interesting as a model outcome.

### Pool stability versus Queue stability

Consider a single differential equation, part of a set of equations that make up a deterministic model,

$$\frac{dX_{i}}{dt}=\gamma_{i}X_{i}-\delta_{i}X_{i}$$ (1)

Trivially, so long as γ and δ are equal to each other, the population of X_i_ is at equilibrium. The population of X_i_ will remain at equilibrium with the addition of arbitrarily many additional terms within the equation so long as these too sum to zero. When extended to the equation system as a whole, the system’s population is in equilibrium so long as

$$\sum_{i=1}^{n}\gamma_{i}X_{i}-\sum_{i=1}^{n}\delta_{i}X_{i}$$ (2)

where *n* is the total number of equations in the system, even if *γ*_*i*_ *X*_*i*_ ≠ *δ*_*i*_ *X*_*i*_ for any particular equation *i* so long as γ and δ are the only parameters that allow the increase or decrease of the system’s population as a whole. However, when these equations are adapted to stochastic simulation, there are two different ways of representing a population steady state that have identical deterministic interpretations, but very different stochastic outcomes.

The first is what I term *“Pool Stability”* and is a direct adaptation of conventional ordinary differential equations. When an algorithm such as Gillespie’s Direct Method [5] is applied to stochastically simulate an ODE-like system without any adaptation, it is this stability mechanism that is implicitly used. Instead of treating γ and δ as deterministic rates of population in-flow and out-flow, they are treated stochastically–the overall rate at which *an* event happens. Which event happens is determined probabilistically based on their relative frequencies [5], in this case set to be equal. In essence this posits an infinite pool of potential members of the population from which members are drawn and to which members return when they exit the system. While these deterministic versus stochastic rates might appear to be direct analogs, they will not necessarily have equal outcomes in the small N populations for which stochastic simulations are often conducted. In the stochastic implementation, the population, despite having members entering and leaving the system at equal rates, may grow above its theoretical equilibrium point, drop below it, or, in some cases, reach zero–a stochastic extinction event.

An alternate strategy for handling stochastic steady states is what I will refer to as *“Queue Stability”*. In this scenario, rather than setting γ and δ to be equal to each other, in- and out-rates are dispensed with entirely. Instead the model incorporates a series of rates that govern when a member of the populations “exit” from the model, whereupon they are immediately replaced by a new individual. This method posits that exiting members of the population free up a space within the model that is then filled by a new member drawn from an infinite queue, rather than being replaced at an equal rate. While not a direct adaptation of the deterministic form of the model, this method stays faithful to the intent of the steady-state terms–the maintenance of a constant population of fixed size.

The latter method seems to eliminate one of the great strengths of stochastic simulation models, the possibility for stochastic extinction of the population, in exchange for maintaining a constant population. This is only partially accurate. While the extinction of the entire model system because of demographic stochasticity is no longer possible, extinction may occur in any given component of the model. This difference gives rise to the need to disambiguate two different potential types of stochastic extinction.

### N- and C-extinction

Two unique types of stochastic extinction events are possible within the compartmental model framework. The first, which is allowed by Pool stability models but prohibited in Queue stability models, is the complete extinction of the model system, where the sum of all compartments is 0, which I term N-extinction[6,7]. The second form of stochastic extinction, which is possible with either form of stability, is the extinction of one of several compartments or subpopulations within the model without the extinction of the entire model population. This phenomenon, which I term C-extinction, is less dramatic but may be of central interest to many research questions[8–12]. For example, the vast majority of epidemiological models are more concerned with the population of infected individuals within a model than with the state of the modeled population as a whole[13].

Like different mechanisms for modeling population mixing and interaction, or changes in model structure, the choice of stabilization method is a fundamental choice about the biological process being modeled, namely whether or not it is possible, within the scope of the model, for the population as a whole to go extinct. For some models, the answer is “Yes”, especially for the small populations most frequently modeled using stochastic techniques, but for other models allowing N-extinction may be irrelevant or actively detrimental to the model’s mapping with reality.

Two models are used as motivating examples to explore this choice and under which conditions one might with to disallow *N-extinction* within their model system. The first is an ecological model of a predator-prey system where the prey species is a secondary or opportunistic source of resources for a predator with a separate birth and death process. The second is a model of *Clostridium difficile* transmission within an intensive care unit (ICU). These two models were chosen both to show the breadth of modeling questions that must address this issue, and also because in each one of the stabilization methods seems intuitively correct, and the other contrary to reality–though which method is which varies between them.

## Methods

### Predator-prey model

The first example model is a straightforward adaptation of a classical Lotka-Volterra predation model[14] with the addition of a separate birth and death process (μ and ϒ respectively) for the predator species (X) (Eq 3). This separate process can be viewed as emigration and immigration of the predator from a nearby patch in a larger meta-population model, or from a second, abstracted predation system. The prey (Y) exists within the model as a secondary species subject to opportunistic predation (β in the prey and δ in the predators). The prey species has an intrinsic birth rate (α). Predator births and deaths were fixed to a steady state using either Pool or Queue stability, and the outcomes of interest were the average (both mean and median) population size of both the predator and prey species over time, as well as whether or not the predator species, the prey species, or both had gone extinct.

$$\begin{array}{l} \frac{dX}{dt}=\delta XY1=\left(1-\frac{X}{\kappa_{X}}\right)+\mu X\left(1-\frac{X}{\kappa_{X}}\right)-\gamma X\left(1-\frac{X}{\kappa_{X}}\right)\\ \frac{dY}{dt}=\alpha Y\left(1-\frac{Y}{\kappa_{Y}}\right)-\beta XY \end{array}$$ (3)

### Hospital infection model

**Fig 1:**
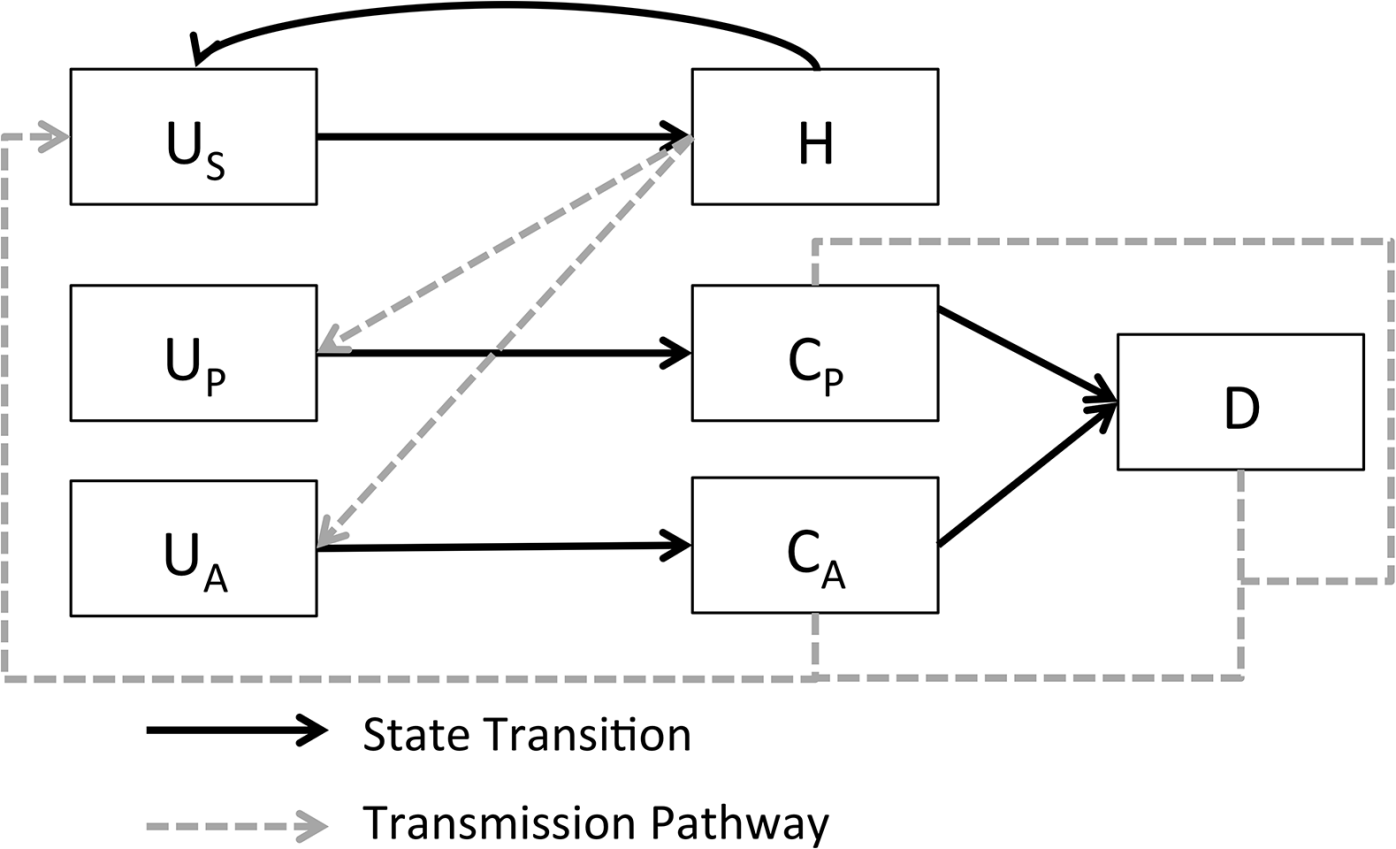
Schematic representation of a mathematical model of within-hospital transmission of *C*. *difficile*, adapted from (4). Healthcare personnel are considered uncontaminated (U_S_) or contaminated (H), and patients are considered to be low risk and uncolonized (U_P_), low risk and colonized (C_P_), high risk and uncolonized (U_A_), high risk and colonized (C_A_) or actively infected (D). Solid arrows denote transitions between states, while dashed lines indicate routes of transmission and contamination between patients and healthcare personnel.

The hospital infection model concerns the spread of *C*. *difficile* within a 12-bed ICU and is based on a previously published model[15] (see also [16–18]). Healthcare personnel were modeled as either uncontaminated (U_S_) or contaminated (H), representing hands or gloves contaminated by *C*. *difficile*. Patients were modeled in a number of treatment and infection states. U_P_ and U_A_, represent patients uncolonized with *C*. *difficile* at low and high risk of infection, respectively, based on whether or not they were on proton pump inhibitors. C_P_ and C_A_ similarly represented colonized patients in low- and high-risk states. Finally, D denoted patients who had developed an active *C*. *difficile* infection (Fig 1 and Eq 4). For specific parameter meanings and values see [15].

$$\begin{array}{l} \frac{dU_{S}}{dt}=\iota H-\rho_{P}\sigma_{P}C_{P}\frac{U_{S}}{N}-\rho_{D}\sigma_{D}D\frac{U_{S}}{N}-\rho_{A}\sigma_{A}C_{A}\frac{U_{S}}{N}\\ \frac{dH}{dt}=\rho_{P}\sigma_{P}C_{P}\frac{U_{S}}{N}+\rho_{D}\sigma_{D}D\frac{U_{S}}{N}+\rho_{A}\sigma_{A}C_{A}\frac{U_{S}}{N}-\iota H\\ \frac{dU_{P}}{dt}=-\rho_{P}\psi_{P}U_{P}\frac{H}{N}-\theta_{P}U_{P}+\nu_{U_{P}}(\theta M+\zeta D+\gamma D)\\ \frac{dU_{A}}{dt}=-\rho_{A}\psi_{A}U_{A}\frac{H}{N}-\theta_{A}U_{A}+\nu_{U_{A}}(\theta M+\zeta D+\gamma D)\\ \frac{dC_{P}}{dt}=\rho_{P}\psi_{P}U_{P}\frac{H}{N}-\kappa C_{P}-\theta_{P}C_{P}+\nu_{C_{P}}(\theta M+\zeta D+\gamma D)\\ \frac{dC_{A}}{dt}=\rho_{A}\psi_{A}U_{A}\frac{H}{N}-\phi C_{A}-\kappa \tau C_{A}-\theta_{A}C_{A}+\nu_{C_{A}}(\theta M+\zeta D+\gamma D)\\ \frac{dD}{dt}=\kappa C_{P}+\kappa \tau C_{A}+\nu_{D}(\theta M+\zeta D+\gamma D)-\zeta D-\gamma \omega D-\gamma (1-\omega )D\\ M=U_{A}+U_{p}+C_{A}+C_{p}+D\\ N=U_{s}+H+U_{A}+U_{p}+C_{A}+C_{p}+D \end{array}$$ (4)

All patients are eventually discharged from the hospital with one of three possible outcomes: discharge from the hospital in good health, discharge from the hospital with the subsequent development of a recurrent *C*. *difficile* infection, and death. Admissions were set to be equal to discharges using either Pool or Queue stability. The outcomes of interest were incident infections within the hospital and discharges that go on to develop recurrent infections, as well as whether or not the patient population went extinct.

### Statistical analysis

The distribution of model outcomes for each model (population medians of predator and prey species and recurrent and incident cases of *C*. *difficile*, respectively) were compared using a two-way Kruskal-Wallis Rank Sum Test, comparing 10,000 model runs, 5,000 each with Pool versus Queue stability. Whether or not the proportion of model runs resulting in a population extinction event was drawn from the same distribution with Pool versus Queue stability was evaluated using a χ^2^ test. The stochastic results are also compared to the models’ deterministic ODE analog, to evaluate whether either approach necessarily has the ODE’s result as its average.

Finally, the mean and median model run times (measured in seconds) were taken for a subset of 100 iterations of each model using both pool and queue equilibrium. All models were implemented in Python using the StochPy library (Version 1.1)[19], and the statistical analysis was performed in R (Version 2.15). Source code and data for the simulations is available at http://dx.doi.org/10.6084/m9.figshare.1047825.

## Results

### Predator-prey model

**Fig 2:**
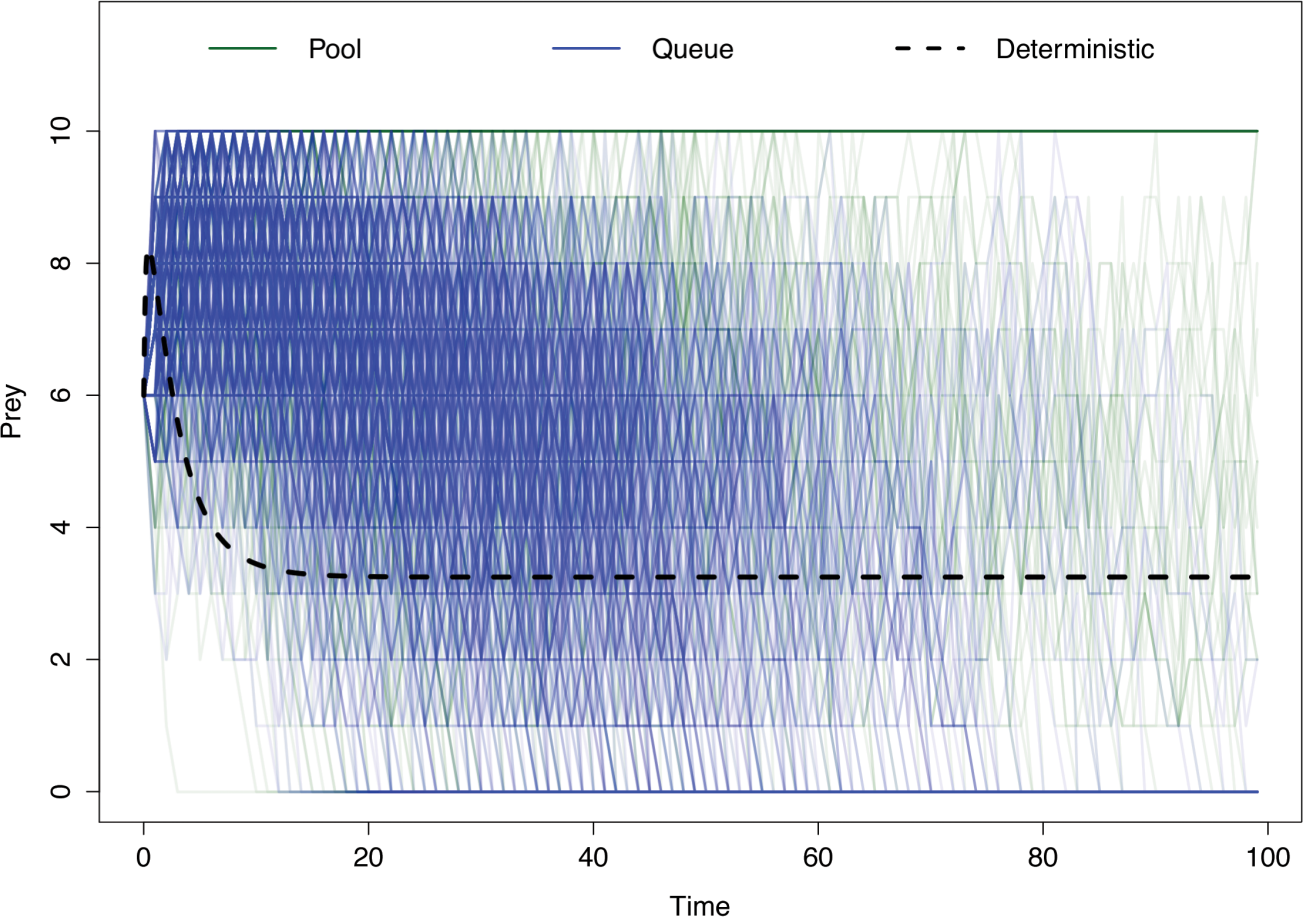
Population trajectories for prey populations for a 10% sample of iterations of Pool and Queue stabilized predator-prey models. Green lines depict the trajectories of Pool stabilized simulations, while blue lines indicate the trajectories of Queue stabilized simulations. All lines are semi-transparent, with areas of more opaque color indicating more frequent results. Dashed line indicates the trajectory of an identically parameterized deterministic model.

**Fig 3:**
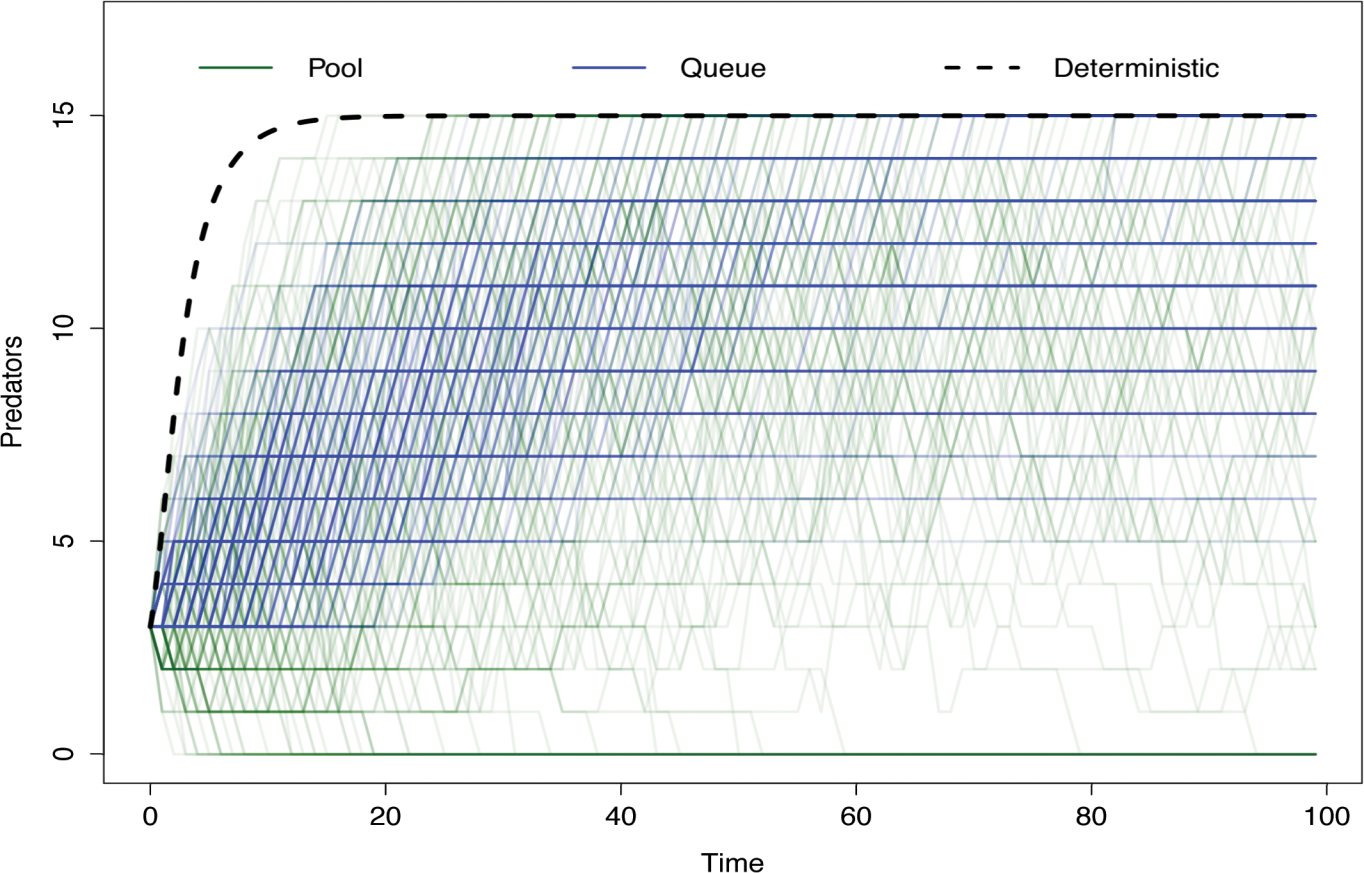
Population trajectories for predator populations for a 10% sample of iterations of pool- and Queue stabilized predator-prey models. Green lines depict the trajectories of Pool stabilized simulations, while blue lines indicate the trajectories of Queue stabilized simulations. All lines are semi-transparent, with areas of more opaque color indicating more frequent results. Dashed line indicates the trajectory of an identically parameterized deterministic model.

The mean and median population for the predator and prey populations, as well as the probability of either species or the entire system going extinct, is reported in Table 1. Queue stability based simulations had higher median and mean predator populations, though the latter was not statistically significant (*P* > 0.001 and p = 0.347 respectively), and correspondingly smaller median and mean prey populations (*P* >0.001 for both measures). While the median prey population in both scenarios was 0, the Pool stable models had a higher mean value (4.06 animals) due to a longer tail of the prey population distribution arising from a small number of scenarios (411) where the predator species experienced stochastic extinction before the prey species, such that the growth of the prey species was then bounded only by the carrying capacity of their habitat. Queue stable models prevent the predator species from going extinct, making such unrestrained population growth impossible for the prey species (mean = 2.93 animals). As it is difficult to summarize stochastic modeling results purely through statistical moments[20], a 10% subsample of population trajectories for both predator and prey populations under each stability mechanism are shown in Figs 2 and 3.

**Table 1:** Results of 5,000 runs of a Pool and Queue Stabilized Predator-Prey Model.

	Pool Stable	Queue Stable	Difference (95% CI)* ^†^	*P*-value
Mean Predators	8.58	9.79	-1.21	0.347
Median Predators	10.50	11	-0.50	>0.001
Mean Prey	4.06	2.93	1.13	>0.001
Median Prey	0	0	0	>0.001
Mean Total Population	12.64	12.72	-0.08	0.001
Median Total Population	13.5	12	1.50	>0.001
Probability of Predator Extinction	0.19	0.00	0.19 (0.18,0.21)	>0.001
Probability of Prey Extinction	0.80	0.99	-0.19 (-0.21, 0.17)	>0.001
Probability of Total Extinction	0.03	0.00	0.03 (0.02,0.03)	>0.001

*95% CI: 95% Confidence Interval.

^†^Confidence intervals not calculated for Kruskal-Wallis tests.

### Hospital infection model

**Fig 4:**
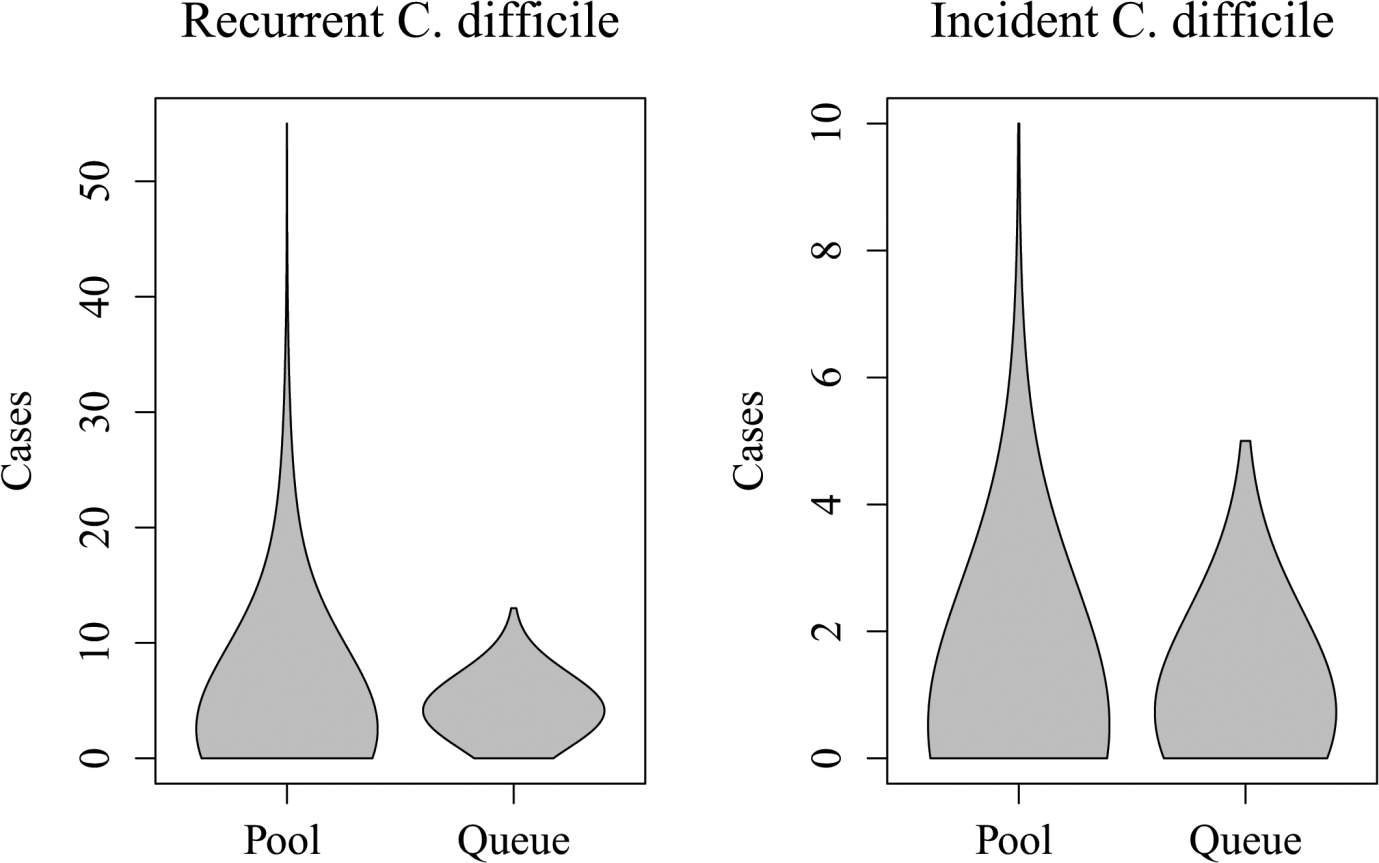
Violin plots of the number of recurrent and incident *C*. *difficile* cases in 5,000 runs of Pool and Queue stabilized stochastic, within-hospital transmission models. Each ‘violin’ represents a smoothed kernel-density estimation of the distribution of cases, mirrored along the y-axis.

The cumulative number of incident and recurrent infections, as well as the probability of the patient population going extinct, is reported in Table 2. Queue stabilized simulations of the hospital system were vastly less likely to experience stochastic extinction, with 71.2% of Pool stabilized model runs resulting in N-extinction, compared to 0.0% of Queue stabilized runs, where such extinction is impossible (*P* > 0.001). As a consequence of more stable patient populations, Queue stabilized models had higher mean and median numbers of cumulative incident and recurrent *C*. *difficile* cases (*P* > 0.001 for all tests). The distributions of cumulative incident and recurrent cases under both stabilization mechanisms are shown in Fig 4.

**Table 2:** Patient Outcomes from 5,000 Runs of a Pool and Queue Stabilized *C*. *difficile* Within-hospital Transmission Model.

	Pool Stable	Queue Stable	Difference	95% CI*	*P*-value
Probability of N-extinction	0.71	0.00	0.71	0.69, 0.73	>0.001
	Pool Stable		Queue Stable		
	Mean	Median	Mean	Median	*P*-value
Incident Cases	0.72	0	0.86	1	>0.001
Recurrent Cases	4.13	2	4.24	4	>0.001

*95% CI: 95% Confidence Interval.

### Comparison to deterministic implementations

Both stochastic predator-prey model implementations had median predator populations well below the deterministic model’s equilibrium state of 15, with the Queue stabilized model’s median predator population of 9.79 slightly closer than the Pool stabilized model’s median of 8.58. Both stochastic models had a median prey population of 0 compared to the deterministic model’s 3.5 animals. The mean prey population of the deterministic model (3.43) fell between the Pool stabilized model’s mean of 4.06 and the Queue stabilized model’s mean of 2.93

The Queue stabilized hospital infection model more closely resembled the deterministic model’s 0.83 incident and 4.22 recurrent cases, with a median of 1 incident and 4 recurrent cases compared to the Pool stabilized model’s 0 incident and 2 recurrent cases. The mean values for incident and recurrent cases for both stabilization types were closer to the deterministic results (see Table 2), but the Queue stabilized model remained more similar.

### Computational time and limitations

**Fig 5:**
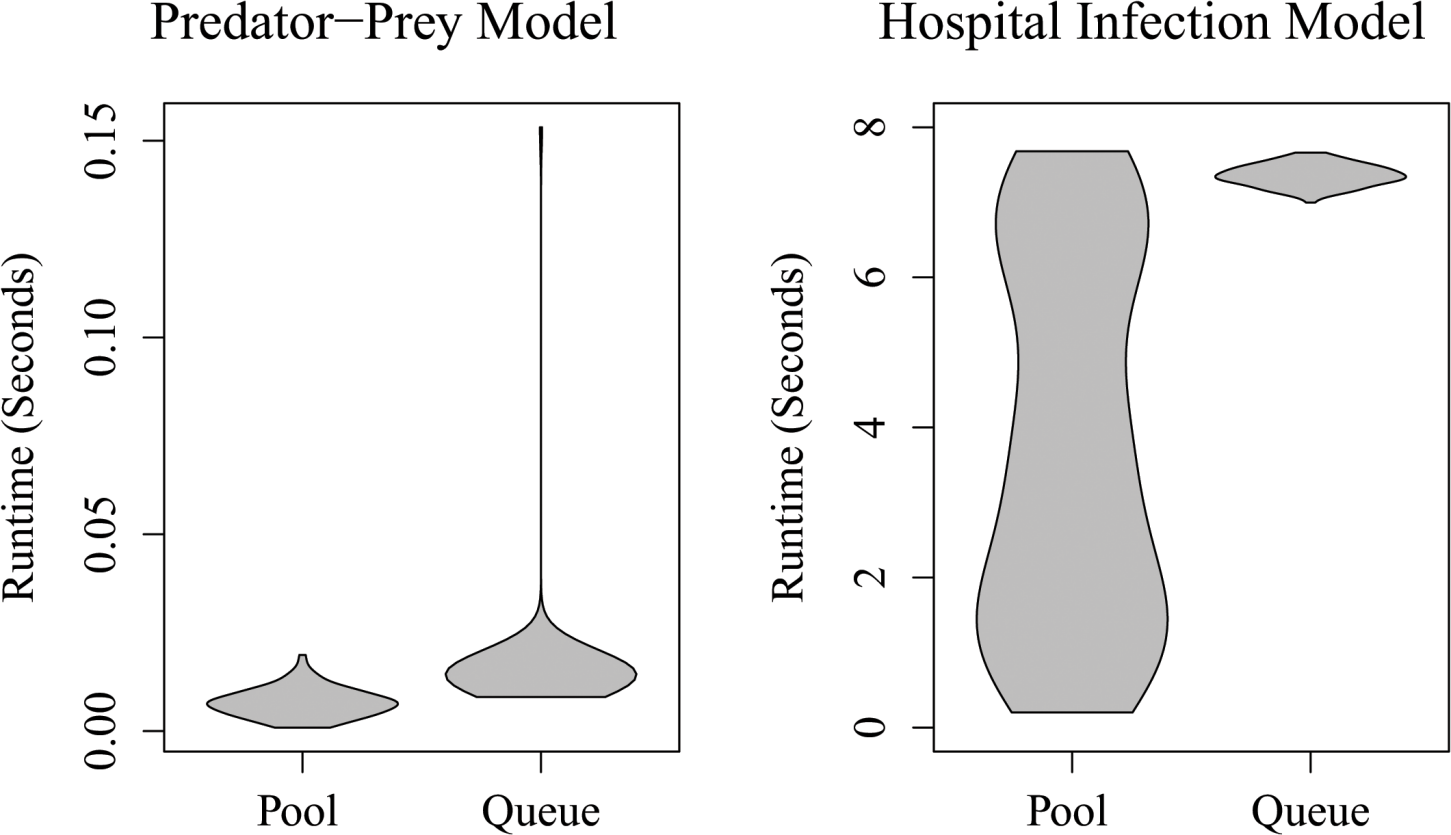
Violin plots of the execution time (in seconds) for a single run of Pool or Queue stabilized predator-prey and *C*. *difficile* within-hospital transmission models. Each ‘violin’ represents a smoothed kernel-density estimation of the distribution of runtimes, mirrored along the y-axis.

For both the predator-prey and epidemic models, the Queue stabilized implementations had a markedly longer runtime, each requiring approximately twice the computation time per iteration when run on an identical 3.2 GHz Intel Core i5 system. The predator-prey model had a median per-iteration runtime of 0.007 seconds for the Pool stabilized version and 0.014 seconds for the Queue stabilized version (*P* < 0.001). The hospital model had a median per-iteration runtime of 3.76 seconds for the Pool stabilized model and 7.36 seconds for the Queue stabilized model (*P* < 0.001). The overall shapes of the runtime distributions were also quite distinct based on which method was used, and the resulting change in the model’s dynamics (Fig 5).

## Discussion

The choice of how one approaches the stochastic modeling of steady-state systems can profoundly influence the results of otherwise identically implemented and parameterized models. Some outcomes, such as the stochastic extinction of an entire system, move from impossible to relatively common. The stability of a system is often considered a “given” within a modeled scenario, rather than a deliberate choice. These differences have the potential to obfuscate results of more direct interest or inject additional disagreement between two different models that predict the same system dynamics but have different equilibrium schemes.

Neither Pool nor Queue stability is inherently superior. The results of the two example models illustrate that the choice of stabilization method is not a straightforward algorithm. In the hospital infection model, Queue stability more closely resembles the results of a deterministic model because it disallows N-extinction. Yet in the predator-prey model, both model implementations differ from their deterministic analog, and in one case the disagreement between them is equal in magnitude and opposite in direction. Despite the Pool stabilized models being seemingly direct adaptations of deterministic ODE models in fact neither stability method clearly mirrors a deterministic model. The only clear, unambiguous difference is the longer computing time for Queue stabilized models.

Instead, the nature of the biological process being modeled should dictate the choice of stabilization method. Specifically, consider whether or not allowing N-extinction is realistic and desirable. For example, it is easy to see how a small wildlife population might be subjected to N-extinction due to string of random events all of which negatively impact survival, and thus how pool-stabilization is an appropriate means to reflect a population at a stochastic steady state. In contrast, Queue stabilization implies something like an infinite line of predators patiently waiting their turn to begin hunting and breeding–an implausible situation suggesting a poor fit for Queue stabilization.

However, despite similarly small population size, intensive care units in major hospitals are relatively stable, and closure to random fluctuations in patient demand is highly unlikely. As such, disallowing N-extinction through a Queue stabilization mechanism is a reasonable choice for modeling such a system. It is significantly easier to picture a line of patients waiting to be admitted into an ICU–indeed, this situation confronts the medical system every day.

This is not an exhaustive study of the effects of how stochastic steady states are modeled, nor even a comprehensive description of all steady-state mechanisms. It is meant to illustrate the importance of considering how each term within a model, even those not of research interest that might be easily overlooked translate to reality, and how reasonable that translation is.
